# The V410L knockdown resistance mutation occurs in island and continental populations of *Aedes aegypti* in West and Central Africa

**DOI:** 10.1371/journal.pntd.0008216

**Published:** 2020-05-08

**Authors:** Constância F. J. Ayres, Gonçalo Seixas, Sílvia Borrego, Cátia Marques, Inilça Monteiro, Camila S. Marques, Bruna Gouveia, Silvania Leal, Arlete D. Troco, Filomeno Fortes, Ricardo Parreira, João Pinto, Carla A. Sousa

**Affiliations:** 1 Global Health and Tropical Medicine, GHTM, Instituto de Higiene e Medicina Tropical, IHMT, Universidade Nova de Lisboa, UNL, Lisbon, Portugal; 2 Department of Entomology, Aggeu Magalhães Institute, Oswaldo Cruz Foundation, Recife, Brazil; 3 Instituto de Administração da Saúde IP-RAM, Secretaria Regional de Saúde e Proteção Civil, e Interactive Technologies Institute, LARSyS, Funchal, Região Autónoma da Madeira; 4 Instituto Nacional de Saúde Pública, Ministério da Saúde e Segurança Social, Praia, Cabo Verde; 5 Direção Nacional de Saúde Pública, Ministério da Saúde, Luanda, Angola; Universita degli Studi di Pavia, ITALY

## Abstract

The extensive use of insecticides for vector control has led to the development of insecticide resistance in *Aedes aegypti* populations on a global scale, which has significantly compromised control actions. Insecticide resistance, and its underlying mechanisms, has been investigated in several countries, mostly in South American and Asian countries. In Africa, however, studies reporting insecticide resistance are rare and data on resistance mechanisms, notably knockdown resistance (*kdr*) mutations, is scarce. In this study, the recently described V410L *kdr* mutation is reported for the first time in old world *Ae*. *aegypti* populations, namely from Angola and Madeira island. Two additional *kdr* mutations, V1016I and F1534C, are also reported for the first time in populations from Angola and Cape Verde. Significant associations with the resistance phenotype were found for both V410L and V1016I individually as well as for tri-locus genotypes in the Angolan population. However, no association was found in Madeira island, probably due to the presence of a complex pattern of multiple insecticide resistance mechanisms in the local *Ae*. *aegypti* population. These results suggest that populations carrying the same *kdr* mutations may respond differently to the same insecticide, stressing the need for complementary studies when assessing the impact of *kdr* resistance mechanisms in the outcome of insecticide-based control strategies.

## Author summary

One of the pillars for the prevention of *Aedes-*transmitted arboviral infections has been vector control, which is primarily based on the use of chemical insecticides. However, extensive use of insecticides has led to the development of insecticide resistance, undermining the sustainability of control programs. Mutations in the voltage-gated sodium channel gene have been associated with knockdown resistance in many insect species including *Aedes aegypti*. In Africa, in spite of the use of insecticides for vector control in many countries, data on insecticide resistance and its underlying mechanisms remain scarce. In this study, we report for the first time the occurrence of a recently described *kdr* mutation, V410L, in Old World *Ae*. *aegypti* from Angola and Madeira island. Two other *kdr* mutations, V1016I and F1534C, were also identified in populations from Angola and Cape Verde, extending our knowledge about the distribution of these mutations in Africa. We found significant associations between *kdr* genotypes and the resistance phenotype but only in the Angolan population. These results suggest that populations carrying the same *kdr* mutations may respond differently to the same insecticide, stressing the need for complementary studies when assessing the impact of *kdr* resistance in the outcome of insecticide-based vector control.

## Introduction

Vector control has been a mainstay for preventing diseases caused by arboviruses transmitted by *Aedes aegypti*, and for mitigating the impact of these infections on human populations. During an outbreak, insecticide-based vector control interventions are usually intensified to reduce mosquito abundance and interrupt human-vector contact. However, increased use of insecticides may result in the selection of mosquitoes carrying genetic traits associated with insecticide resistance. The emergence of insecticide resistance in natural vector populations can ultimately affect the efficacy of insecticide-based vector control.

Target site insensitivity is a major mechanism of insecticide resistance that results from point mutations in genes encoding proteins at the specific site where an insecticide binds, typically in the nervous system [1]. These mutations cause structural modifications that reduce or even completely block the binding of the insecticide. The voltage-gated sodium channel (VGSC) is the target site of pyrethroid and organochlorine (notably DDT) insecticides. Mutations in the gene encoding the VGSC have been implicated in insecticide resistance in several insect species, a phenomenon referred to as knockdown resistance (*kdr*) [2]. In *Ae*. *aegypti*, a total of 10 *kdr* mutations have been reported [1]. Among these mutations, the V1016I and F1534C mutations have been extensively investigated in pyrethroid-resistant *Ae*. *aegypti* populations from Asia, South America and, to a lesser extent, Africa [2].

In 2017, a novel *kdr* mutation, V410L, located in domain I of segment 6 of the VGSC, was described for the first time in a pyrethroid-resistant *Ae*. *aegypti* laboratory strain originating from Rio de Janeiro, Brazil [3]. Functional analysis of mutant sodium channels expressed in *Xenopus* oocytes revealed that V410L significantly reduced sensitivity to both type I and type II pyrethroids. Genotyping of natural populations from Northeast Brazil did not reveal the presence of this mutation [3]. However, in a longitudinal study carried out in Mexico, the V410L mutation was detected in a single heterozygous mosquito in 2002 and by 2012 its frequency had increased to 90% in one of the localities surveyed [4]. The same mutation was described in natural populations of *Ae*. *aegypti* from Colombia, with frequencies ranging from 0.06 to 0.36 [5].

In recent years, Africa has been experiencing major outbreaks of *Aedes-*borne arboviral infections. Such was the case for a dengue outbreak that occurred in Cape Verde, in 2009, which affected eight of the 10 islands of this archipelago with more than 21,000 cases reported and four deaths [6]. In 2015–2016, Angola (South-Central Africa) experienced the worst epidemic of yellow fever in the last 60 years, with 4,306 notified cases and 376 deaths occurring in the province of Luanda [7]. The country had previously been afflicted by a Dengue outbreak in 2013 (https://www.ncbi.nlm.nih.gov/pubmed/23784016). In addition to the above mentioned outbreaks, Angola and Cape Verde were also affected by Zika outbreaks in 2015 and 2016, and cases of congenital Zika syndrome causing microcephaly were reported in both countries [8–10].

On Madeira Island, a Dengue outbreak involving 2,168 notified cases occurred in 2012–2013 [11]. Although this island is located *ca*. 680 km east off the coast of Morocco, it belongs administratively to Portugal and the European Union, raising concern about the risk of the introduction of both vectors and viraemic cases to mainland Europe.

As a response to these outbreaks, vector control programs tend to intensify insecticide-based interventions as a means of rapidly halting arbovirus transmission. This may lead to emergence of insecticide resistance in the local vector populations. Insecticide susceptibility tests carried out in 2009 on *Ae*. *aegypti* from the city of Praia, the capital of Cape Verde, revealed resistance to DDT but susceptibility to all other insecticides tested [12]. Subsequently, a survey carried out in 2012 and 2014 in the same urban area revealed resistance to deltamethrin and cypermethrin, but no *kdr* alleles were detected [13]. The local *Ae*. *aegypti* population of Madeira Island was found to be resistant to all major insecticide classes currently used in public health practices. This population displays multiple mechanisms of resistance, including the presence of V1016I and F1534C *kdr* mutations at a high frequency [14].

In Africa, information on mechanisms of insecticide resistance in *Ae*. *aegypti* populations remains scarce [2]. The presence of both F1534C and V1016I has been detected in Ghana [15] and in Burkina Faso [16], although it has also been investigated in Cameroon [17], Cape Verde and the Central African Republic [18]. None of the previous reports have analysed the new V410L *kdr* mutation. Considering that this mutation has shown potential to significantly reduce sodium channel sensitivity to both type I and II pyrethroid and to increase resistance in combination with F1534C [3], it is imperative to determine the current distribution of this mutation in natural *Ae*. *aegypti* populations, as its presence may greatly undermine the utility of a wide variety of pyrethroid insecticides, which are currently the major insecticide class used in vector control.

In this study, we genotyped the *kdr* loci of *Ae*. *aegypti* populations from Angola, Cape Verde and Madeira Island, collected either during or after the onset of arboviral outbreaks. The objectives were: i) to determine the presence and frequency of the V410L, V1016I and F1534C mutations and ii) to investigate the association of these mutations with the pyrethroid and DDT resistance phenotype.

## Material and methods

### Samples

**Fig 1:**
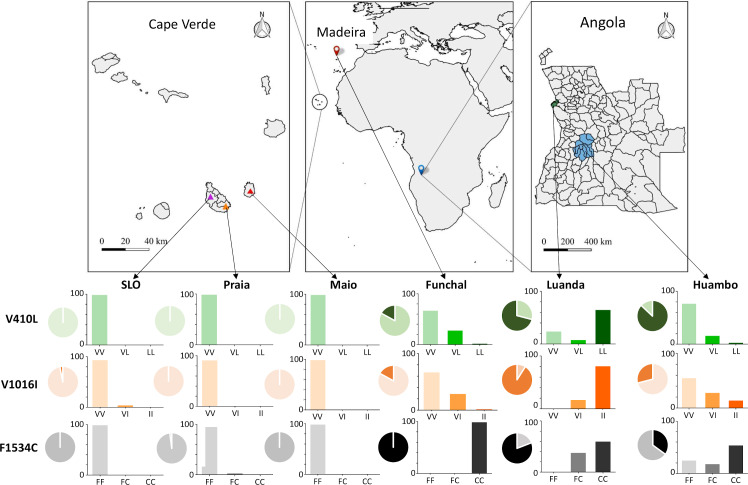
Collection sites, allele frequencies (pie charts) and genotypic frequencies (bar charts) for each of the *kdr* mutations analysed. In each allele frequency pie chart, light colour represents the wild-type (susceptible allele) and dark colour the resistance-associated allele. Map source: edited in QGIS v.3.8.0 (July, 2019).

In Cape Verde, mosquito collections were carried out from September 2017 to March 2018 in two islands: Santiago and Maio. In Santiago, mosquitoes were sampled in Praia (N 14º 55’ 15” W 23º 30’ 30”), the capital of the country, and São Lourenço dos Órgãos (SLO) (N 15º 6’ W 23º 60’). In Maio, sampling was performed in the main city, Maio Village (N 15º 13’ W 23º 10’). In Angola, collections took place in October-December 2016, in the cities of Huambo (S 12º 46’ E 15º 44’) and the capital, Luanda (S 8º 50’ 18” E 13º 14’ 4”). On Madeira island, mosquitoes were collected in Funchal (N 32º 39’ W 16º 55’) between September and November 2013 [14] (Fig 1).

In all but one of the localities surveyed, mosquito eggs were collected using ovitraps, adapted from the model of Faye & Perry [19]. Ovitraps were placed outdoors at peridomestic sites for 5–7 days. In Funchal, eggs were collected using 78 ovitraps dispersed throughout the entire municipality below 200 meters of altitude [14]. These ovitraps are part of Madeira’s *Aedes aegypti* surveillance program and their geo-localization is available at http://iasaude.sras.gov-madeira.pt/naomosquito/. In Cape Verde, collections were performed with 40 ovitraps distributed to cover most of the area of each locality surveyed. Collections in Luanda were carried out using 49 ovitraps covering an area of *ca*. 70 km^2^ in the Great Luanda region. Larval collections with dips and pipettes were performed in two areas of the city of Huambo, the city centre and one semi-rural area.

Collected eggs (or larvae) were transported to local laboratory facilities and reared to adults using standard protocols for mosquito rearing [20].

### Insecticide resistance phenotyping

Mosquito samples from Cape Verde (Maio and Santiago islands), Madeira island and from Luanda-Angola were phenotyped for their susceptibility to deltamethrin 0.05% and permethrin 0.75% using WHO test kits and protocols [21,22]. In Madeira, cyfluthrin (0.15%) was tested instead of deltamethrin. Non-blood fed 3–5 days-old females were used in the tests. Bioassay readings were made 24 hours post-exposure. Dead mosquitoes were considered susceptible while survivors were considered resistant. Both were kept in silica-gel filled tubes until DNA extraction.

#### Kdr genotyping

**Fig 2:**
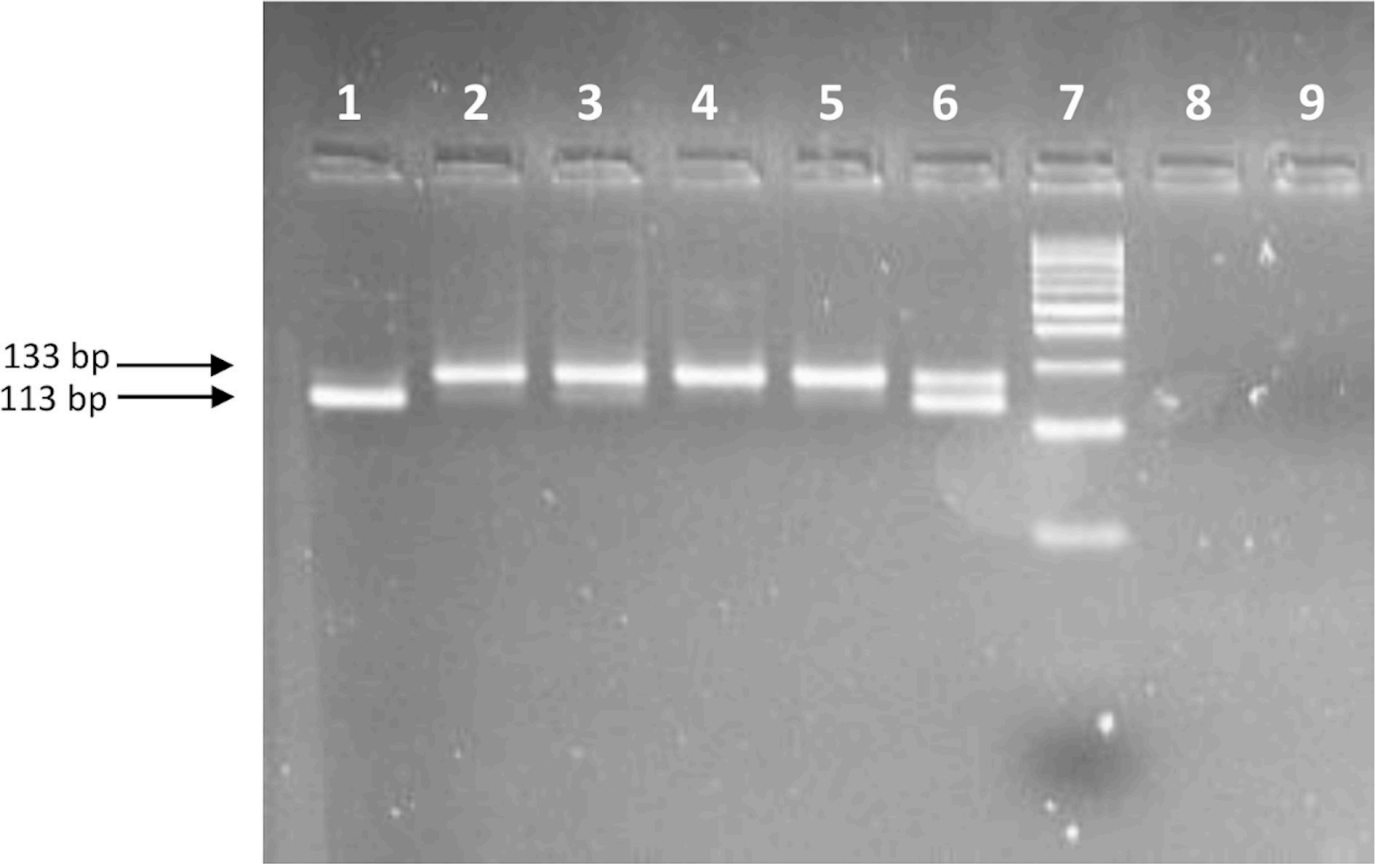
Agarose gel (4%) stained with GreenSafe Premium containing the amplified products of the AS-PCR assay to detect the V410L mutation. **Lane 1: homozygous 410L genotype, lanes 2–5: homozygous 410V genotype, lane 6: heterozygous 410V/L genotype, lane 7: DNA size marker GRS DNA Ladder 50 bp (GRiSP Research Solutions, Portugal)**. Lanes 8–9: negative controls.

DNA samples were extracted from individual whole mosquitoes using the Collins protocol [23]. DNA pellets were eluted in 200 μL of ultrapure water. Genotyping of the V1016I and F1534C mutations was performed by allele-specific PCR using previously reported primers and protocols [24,25], modified as described by Seixas et al. [14]. For V410L, two protocols were used: 1) Direct sequencing of a 150 bp fragment of the *vgsc* gene amplified using the primers Ae410F1 and Ae410R1 described in [3]. PCR assays were carried out in a 25 μl volume containing: 1x Green Flexi PCR buffer, 1.5 mM MgCl_2_, 0.4 Mm dNTP mix, 100 nM of each primer, 1 unit GoTaq G2 Flexi DNA Polymerase (Promega, USA) and 10 ng template DNA. The cycling conditions consisted of denaturation at 94 °C for 2 min, followed by 35 cycles of denaturation at 94 °C for 40 s, primer annealing at 58 °C for 45 s, and extension at 72 °C for 1 min, with a final extension of 10 min at 72 °C. The PCR products were purified using Antarctic phosphatase (NEB) and Exonuclease I (NEB) to remove unincorporated dNTPs and primers. The purified PCR products were diluted 1:30 and subjected to direct sequencing in an ABI Prism 3500XL system at the DNA sequencing platform of the Aggeu Magalhães Institute/FIOCRUZ (Brazil) or in an ABI 3730 XL instrument at the STAB VIDA DNA Sequencing Service (Portugal). Sequencing was performed in both directions using the primers Ae410F2 and Ae410R1 [3]. 2) An allele-specific PCR assay with primers V410Fw, L410Fw and 410Rev described by Saavedra-Rodriguez [4]. The reaction mixtures consisted of ~10 ng of template DNA, 0,1 μM of each primer, 1x Green Flexi PCR buffer, 1.5 mM MgCl_2_, 0.4 Mm dNTP mix, 1 unit GoTaq G2 Flexi DNA Polymerase (Promega, USA) in a total volume of 25 μL. Each PCR assay included positive controls for each homozygous and heterozygous genotypes that had been previously confirmed with direct sequencing and a negative control (no DNA template). PCR products were visualized in a agarose gel 4% stained with Greensafe Premium (NZYTech, Portugal) by transillumination under UV (Fig 2).

### Statistical analysis

To assess the association between *kdr* genotypes and resistance phenotypes, Fisher’s exact test was calculated with contingency tables using VassarStats (Vassarstats: Website for Statistical Computation). Pairwise estimates of linkage disequilibrium coefficients and associated chi-squared tests between loci were calculated using LINKDOS [26], following previously described guidelines [4,27].

## Results

In total, 314 *Ae*. *aegypti* were genotyped, 91 of which were from Madeira Island, 168 from Cape Verde (80 from Praia, 39 from São Lourenço dos Órgãos (SLO) and 49 from the island of Maio) and 95 from Angola (67 from Luanda and 28 from Huambo). Of the total sample, mutation V410L was screened by direct sequencing in 91 individuals from Madeira, 67 from Cape Verde and 95 from Angola. Sequences obtained from *Ae*. *aegypti* mosquitoes from Madeira are available in GenBank with accession numbers MK656133-MK656223. Apart from the V410L, no other genetic polymorphism was detected in the 104 bp fragment analysed for Madeira population. The genotype and allele frequencies of each *kdr* mutation are shown in Fig 1. The 410L allele was not observed in Cape Verde. The resistance allele 1534C was detected at a low frequency (2%) in Praia, the capital, always in heterozygosity with the wild type allele 1534F. Likewise, the 1016I allele was found in two heterozygous individuals from the SLO population. In Angola, allele 410L was observed at a high frequency in Luanda (71%) and at moderate frequency in Huambo (13%). Allele 1534C was observed at high frequency in both cities of Angola, while allele 1016I was detected at much higher frequency in Luanda (91%) than in Huambo (29%) (Fig 1). On Madeira Island, the 410L allele was observed at a moderate frequency (18%). As previously reported [14,28], the 1534C mutation was found to be fixed, and 1016I was present at a moderate frequency.

Pairwise linkage disequilibrium analysis revealed significant genotypic associations between positions 1016 and 410 in all populations tested (Table 1). For positions 1016 and 1534, only the Huambo population showed significant linkage disequilibrium. Finally, positions 1534 and 410 were in linkage equilibrium in all three populations tested. This analysis was not performed in Cape Verde due to the almost complete absence of mutant alleles.

**Table 1:** Linkage disequilibrium coefficients (*R*_*ij*_) and associated chi-squared tests between *kdr* mutations in *Aedes aegypti* populations.

	410–1016			410–1534			1016–1534		
	*R*_*ij*_	*χ^2^*	*P*	*R*_*ij*_	*χ^2^*	*P*	*R*_*ij*_	*χ^2^*	*P*
Luanda	0.19	4.53	0.033	0.18	2.94	0.087	0.07	0.25	0.619
Huambo	0.33	7.67	0.006	0.22	2.74	0.098	0.29	5.15	0.023
Funchal	0.95	79.65	<0.001	*	*	*	*	*	*

*An allele at one or both loci is fixed

Mortality rates for the insecticides tested varied between regions, being highest in the populations of Cape Verde islands (89.9%-100.0%) and lowest in Luanda-Angola (2.6%-7.4%). These results are detailed in S1 Table. Mortalities of 10.9%-52.8% obtained in Funchal, Madeira island, were intermediate to those of the other two regions, as described in Seixas *et al*. [14].

**Fig 3:**
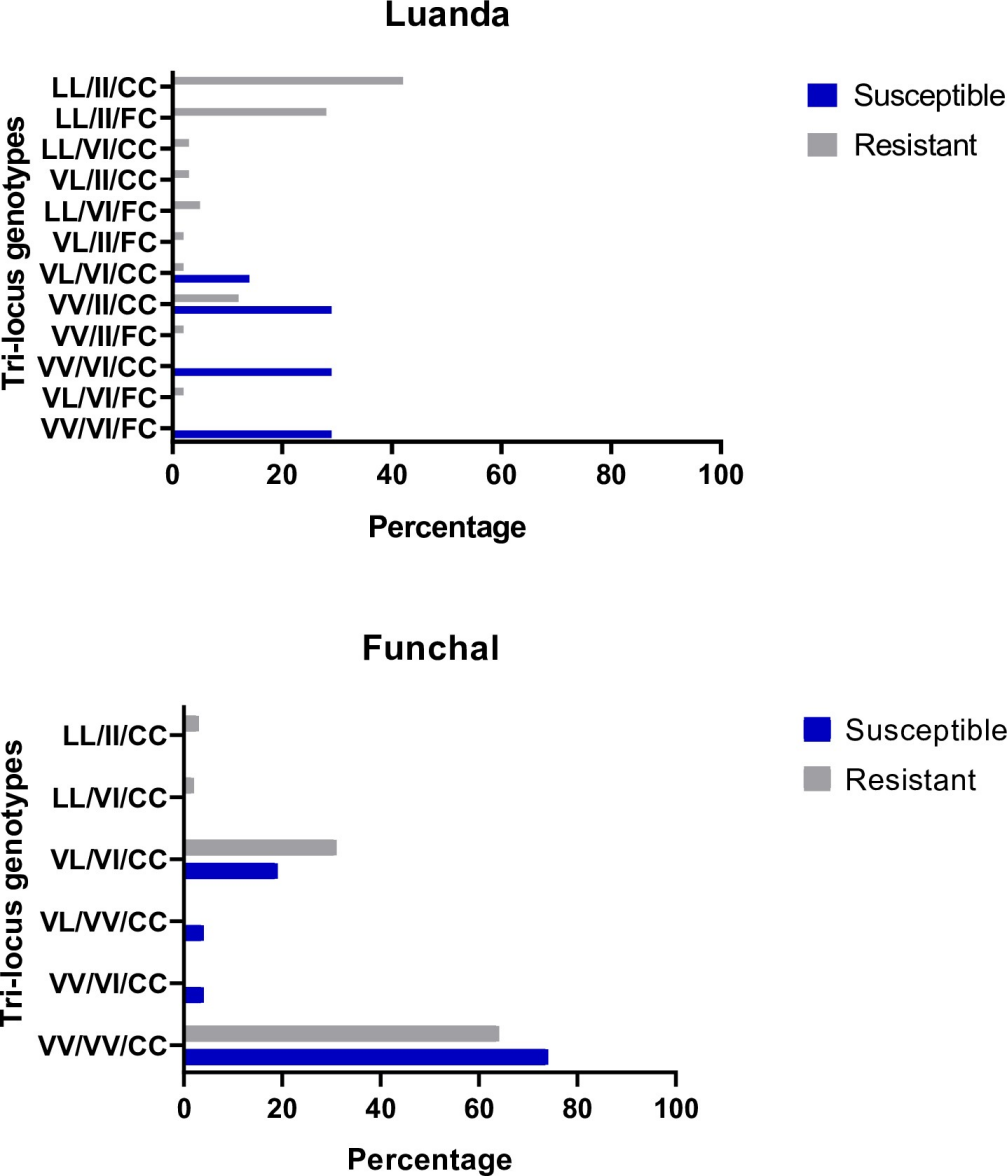
Frequencies of tri-loci genotypes in phenotyped mosquitoes from Luanda and Madeira. Each tri-locus genotyped is named according to the genotypic composition at each *kdr* mutation following the order 410 (VV, VL or LL) / 1016 (VV, VI or II) / 1534 (FF, FC or CC).

For the populations of Madeira and Luanda, it was possible to analyse the association between *kdr* mutations and the resistant phenotype. In Luanda, there were significant associations between resistance phenotypes and genotypic frequencies for mutations V1016I and V410L, but not for F1534C (Table 2). No significant association between phenotypes and individual *kdr* genotypes was found in the population of Madeira. This lack of association in Madeira was also evident for tri-locus genotypes, where the most frequent VV/CC/VV was common to both susceptible and resistant individuals (Fig 3).

**Table 2:** Knockdown resistance genotype-phenotype associations in *Ae*. *aegypti* populations from Luanda (Angola) and Funchal (Madeira island).

		V410L						V1016I						F1534C					
Locality	Phenotype	N	VV	VL	LL	F(L)	*p*-value	N	VV	VI	II	F(I)	*p*-value	N	FF	FC	CC	F(C)	*p*-value
Luanda	Deltamethrin resistant	24	4	0	20	0.83	<0.001	25	0	0	25	1.00	<0.001	25	0	12	13	0.76	0.699
	Deltamethrin susceptible	10	8	2	0	0.10		11	0	6	5	0.73		8	0	3	5	0.81	
Funchal	Cyfluthrin resistant	32	20	9	3	0.23	0.387	32	20	10	2	0.22	0.491	32	0	0	32	1.00	n.a.
	Cyfluthrin susceptible	19	15	4	0	0.11		19	15	4	0	0.11		19	0	0	19	1.00	
	Permethrin resistant	32	21	11	0	0.17	1.000	32	21	11	0	0.17	1.000	32	0	0	32	1.00	n.a.
	Permethrin susceptible	8	6	2	0	0.13		8	6	2	0	0.13		8	0	0	8	1.00	

n.a.: not applicable (fixed mutation)

Conversely, in Luanda, there was a trend for individuals with a resistant phenotype to carry tri-locus genotypes with resistance-associated alleles. Here, the triple mutant homozygote II/CC/LL was present in nearly 50% of resistant individuals.

## Discussion

This study describes for the first time the occurrence of the recently described V410L *kdr* mutation in old world *Ae*. *aegypti* populations, namely from Madeira island and Angola. In both cases, this mutation coexists with mutations F1534C and V1016I, which are widely distributed across the Globe [4]. The V410L mutation was originally described in Central and South American populations of *Ae*. *aegypti*, namely from Brazil, Colombia and Mexico [4,5]. This mutation appears to increase considerably resistance to pyrethroid insecticides when in combination with other *kdr* mutations [3,4]. It is likely that the occurrence of this mutation will have an impact on the efficacy of pyrethroid-based control measures against *Ae*. *aegypti* currently implemented in Africa.

The detection of mutations F1534C and V1016I in two sites from Angola extends our knowledge about *kdr* distribution to southern African populations of *Ae*. *aegypti*. Previous reports revealed the presence of the two mutations only in West African populations of Ghana and Burkina Faso [15,16]. However, the number of *kdr* surveys is still scarce for the African continent (reviewed in [2]) so that absence of records may result from under-sampling rather than true absence of this resistance mechanism. Therefore, it is likely that *kdr* mutations are more widespread in *Ae*. *aegypti* across the African continent than previously thought. Additional *kdr* surveys should thus be prioritised in order to clarify the current distribution of these mutations in African *Ae*. *aegypti* populations.

In addition to mainland Africa, *kdr* mutations were also found in *Ae*. *aegypti* from two outer West African archipelagos, Madeira and Cape Verde. In Cape Verde, mutation V410L was not detected whereas mutations V1016I and F1534C were found at low frequency (≤ 3.0%) in Santiago, the main island of the archipelago. Previous analysis of samples collected in the same island in 2007, 2010 and 2012 did not reveal the presence of any of these mutations [13,29]. This suggests a very recent origin of *kdr* mutations in this island. Whether these mutations represent introductions or independent mutation events is unknown. A recent phylogeographic study suggests that *Ae*. *aegypti* from Cape Verde originated from populations of Senegal [29]. One cannot rule out the possibility of a recent introduction of *kdr* mutations from neighbouring regions of mainland West Africa, specifically of mutation F1534C found in the city of Praia, where an international airport and port are located. On the other hand, the detection of the V1016I mutation in the remote inland locality of São Lourenço dos Órgãos argues in favour of an independent mutation event. The recent emergence of *kdr* mutations in Cape Verde may also explain the low frequency of resistant-associated alleles despite increased insecticide pressures imposed by vector control since the Dengue epidemic of 2009 [30].

In contrast to Cape Verde, the three *kdr* mutations analysed were present in Madeira Island. One of these mutations, F1534C, was fixed while the others displayed moderate frequencies. These *kdr* frequencies do not seem to be fully explained by insecticide pressures, given that vector control in Madeira has been predominately based on the elimination of mosquito breeding sites. A more plausible explanation is that *kdr* frequencies reflect those of the source populations that colonized Madeira Island [31]. Phylogenetic analysis suggests that *Ae*. *aegypti* was recently introduced into Madeira Island from South-American source populations, notably from Venezuela [31]. This region has well-documented insecticide resistant *Ae*. *aegypti* populations displaying high frequency of the F1534C and varying levels of the V1016I mutation [32].

In Luanda, Angola, significant associations were found between resistant-associated *kdr* alleles and the resistant phenotype to deltamethrin. This was evident for tri-locus genotypes and individually for mutations V410L and V1016I. The lack of association of mutation F1534C with resistance is consistent with previous observations suggesting that this mutation alone confers low levels of resistance but co-evolved with V1016I yielding higher levels of resistance [27,33]. Linkage disequilibrium coefficients were also highest between these V410L and V1016I mutations, which may reflect epistatic selection through insecticide pressure. During the 2016 yellow fever outbreak, vector control based on larvicides and indoor/outdoor pyrethroid spraying was intensified in Luanda, which substantially reduced the mosquito population [34]. It should be noted, however, that this is the first *kdr* survey in *Ae*. *aegypti* from Angola, so that no historical data are available for comparison. In Angola, insecticides have been widely used for vector control since the DDT-based malaria eradication campaigns implemented in the 1950s [35,36].

The results obtained in Luanda overall agree with the findings of longitudinal surveys carried out in Mexico, where pyrethroid have been routinely used by operational programs to control malaria and arbovirus vectors since 2000 [4,37]. Coincidently, the frequency of V410L has substantially increased alongside with V1016L and F1534C between 2000 and 2016 and significant associations were found between resistant-associated alleles and pyrethroid-resistant phenotypes [4]. However, the estimates of linkage disequilibrium coefficients (*R*_*ij*_) found in Luanda (0.07–0.33) were generally much lower than those obtained in Mexico (0.31–0.99; [4]), which may reflect an earlier stage of selection of resistant-associated alleles.

In contrast with the observations in Luanda and Mexico, there were no associations between resistance associated *kdr* alleles and pyrethroid resistance in Madeira island. This result was unexpected but it probably reflects the complex pattern of multiple insecticide resistance mechanisms present in the local *Ae*. *aegypti* population [14]. In addition to *kdr*, a recessive trait, microarray-based gene expression analysis provided evidence for metabolic and cuticular resistance mechanisms, which may disrupt the statistical association between phenotypes and *kdr* genotypes. Also noteworthy is that the highest linkage coefficient between V410L and V1016I was found in Madeira island, a result that agrees with the hypothesis of these mutations being introduced in the island from a few colonizing individuals already carrying multiple resistance associated alleles [14].

In summary, we report the occurrence of the V410L *kdr* mutation in populations of *Ae*. *aegypti* from the old world for the first time. As also observed in South America, this mutation appears to coevolve with V1016I providing substantial higher levels of resistance to pyrethroid in the population of Angola. However, this was not the case of Madeira island, where association between *kdr* and pyrethroid resistance is probably disrupted by the coexistence of multiple resistance mechanisms. These findings suggest that populations carrying *kdr* mutations may respond differently to pyrethroid. Further studies will be required to assess the real impact of *kdr* mechanisms in the outcome of insecticide-based control of *Ae*. *aegypti*.

## Supporting information

S1 Table Mortality rates of *Aedes aegypti* from Angola and Cape Verde islands exposed to insecticides at diagnostic doses. (DOCX)
