# Supplementary material for: The V410L knockdown resistance mutation occurs in island and continental populations of Aedes aegypti in West and Central Africa
Source: PLoS Negl Trop Dis. 2020 May 8;14(5):e0008216. doi: 10.1371/journal.pntd.0008216 (PMC7304628; doi:10.1371/journal.pntd.0008216)
Supplement: S1 Table — (DOCX) [file pntd.0008216.s001.docx]

**Table S1. Mortality rates of *Aedes aegypti* from Angola and Cape Verde islands exposed to insecticides at diagnostic doses.** *N*: number of mosquitoes tested; % Mort.: percent mortality.

| Country | Collection site | Permethrin (0.75%) | | Deltamethrin (0.05%) | |
| --- | --- | --- | --- | --- | --- |
|  |  | *N* | % Mort. | *N* | % Mort. |
| Angola | Luanda | 116 | 2.6 | 109 | 7.4 |
| Cape Verde | Maio island | 106 | 100.0 | 106 | 89.8 |
|  | Praia, Santiago island | 113 | 100.0 | 114 | 100.0 |
|  | SLO, Santiago island | 122 | 99.6 | 116 | 100.0 |
